# The association of dietary patterns with muscle mass and strength in old age: The Hordaland Health Study

**DOI:** 10.1007/s00394-023-03206-9

**Published:** 2023-07-11

**Authors:** Zoya Sabir, Jutta Dierkes, Anette Hjartåker, Hanne Rosendahl-Riise

**Affiliations:** 1 Centre for Nutrition, Mohn Nutrition Research Laboratory, Department of Clinical Medicine, University of Bergen, 5020 Bergen, Norway; 2 Department of Nutrition, Institute of Basic Medical Sciences, University of Oslo, Oslo, Norway

**Keywords:** Diet quality, Dietary patterns, Muscle mass, Muscle function, Sarcopenia, The Hordaland Health Study

## Abstract

**Purpose:**

The single nutrient approach in nutrition research lacks the ability to account for synergistic relationships between dietary components. Current evidence suggests that diet quality, reflecting overall dietary intake, may influence muscle health. In a community-based observational study in Western Norway, we examined dietary patterns in relation to muscle mass and strength at age 67–70.

**Methods:**

The current analysis was conducted in men and women of The Hordaland Health Study (HUSK), who participated in both the second (HUSK2) and third study wave (HUSK3). Dietary patterns were extracted by principal component analysis (PCA) on food frequency questionnaire (FFQ) data. Individual dietary pattern scores (DPS) for HUSK2 (age 46–49) and HUSK3 (age 67–70), and overall DPS (oDPS) were calculated. Outcome variables were appendicular skeletal muscle mass (ASMM) and handgrip strength (HGS) measured in HUSK3. The relationships of HUSK3 DPS and oDPS with ASMM and HGS were assessed by multivariate linear regression analysis adjusted for potential confounding factors.

**Results:**

We identified three distinct dietary patterns, labelled ‘Western’, ‘Healthy’, and ‘Sweets-focused’. A significant positive association was observed between the oDPS for the ‘Healthy’ dietary pattern and ASMM in both men and women at age 67–70. No significant associations were found between HUSK3 DPS or oDPS for any of the identified dietary patterns and HGS in our population.

**Conclusion:**

Higher oDPS on a dietary pattern predominantly rich in fish, vegetables, nuts and seeds, fruit and berries, and eggs was associated with better ASMM at age 67–70. To establish the influence of diet quality on muscle health, further long-term studies with repeated dietary assessments are warranted.

## Introduction

The most prominent age-associated physiological changes in musculoskeletal health include loss of muscle mass and muscle strength [1]. Between middle age and the age of 70, muscle mass is estimated to decline by 8% per decade. By the age of 80, this progressive decline may have contributed to a 50% loss of total muscle mass [2]. From middle to older age, upper body strength decreases by an estimated 2–12% per decade, largely independent of the decrease in muscle mass. Despite the modest loss of approximately 1% leg lean mass per year, lower body strength decreases by 3–4% annually [3]. It has been proposed that muscle mass and strength in old age do not merely reflect the rate of loss, but also the peak achieved earlier in life, which typically occurs in young adulthood [4–6]. To decelerate the decline in musculoskeletal health, the aim is thus to maximize the peak of muscle mass and strength attained in adolescence and young adulthood, maintain it in middle age, and minimize loss in older age [4, 7, 8].

Although declines in muscle mass and muscle strength with ageing are regarded as somewhat physiological, the condition becomes pathological below certain thresholds of low muscle mass and strength, and is then termed sarcopenia. Sarcopenia predisposes affected individuals to an increased risk of falls, fractures, physical disability, and mortality [7, 9]. The revised guidelines by The European Working Group on Sarcopenia in Older People (EWGSOP2) recognize low grip strength, rather than muscle mass alone, to be a more powerful predictor of adverse patient outcomes, including longer duration of hospitalization, steeper functional decline, poor health-related quality of life, and mortality [6, 7, 10].

Several mechanisms contribute to the loss of muscle mass and strength, including dietary factors, physical inactivity, hormonal changes, inflammation, increased catabolism, resistance to anabolic stimuli, loss of myocytes, reduced number and function of satellite cells, mitochondrial dysfunction, insulin resistance, and the menopause in women [11–13]. Current evidence highlights the implication of dietary risk factors in the decline of muscle mass and strength, such as dietary protein, amino acids, vitamin D, omega-3 fatty acids, and magnesium [3, 14–16].

While nutritional epidemiology has traditionally focused on exploring the relationship of single nutrients and foods with muscle mass and strength, real-life dietary intake represents combinations and replacements of foods and nutrients [17]. As the conventional approach lacks the ability to account for the synergistic interplay of nutrients and foods in complete diets, dietary pattern analysis is an alternative approach [17, 18]. Dietary pattern analysis facilitates the investigation of the overall diet, while reflecting real-life food choices and eating behaviours to a greater extent [19, 20]. Among the most widely applied à posteriori (exploratory) approaches for dietary pattern analysis is the principal component analysis (PCA) [21], which derives underlying dietary patterns based on the correlation between food groups. PCA makes use of the correlation matrix to reduce a large number of food group variables to a smaller set of principal components that are uncorrelated—and explain as much of the variation in food intake as possible [22].

Previous studies addressing the relationship between overall diet quality and muscle mass and function (muscle strength and physical performance) report conflicting findings, independently of whether diet quality is described using à priori or à posteriori approaches [23]. Another challenge in the investigation of dietary intake and loss of muscle mass and strength is the need to decipher the potential role of diet quality across adulthood. This requires repeated dietary data collections, preferably using identical or similar assessment methods. There is a scarcity of studies using dietary data collected at more than a single time point to investigate dietary patterns in relation to muscle mass and function. In the Hordaland Health Study, dietary data were collected both in middle and old age. Thus, in the current study, we computed overall dietary pattern scores (oDPS) based on dietary data collected in middle age (46–49 years) and old age (67–70 years). We examined the association of oDPS with muscle mass and muscle strength measured at age 67–70 years. Due to the methodological uncertainties related to comparing dietary data collected approximately 20 years apart, we also examined cross-sectional associations of HUSK3 DPS with muscle mass and muscle strength at age 67–70 years.

## Methods

### Study sample

The current study was conducted in participants from the community-based Hordaland Health Study (HUSK) (https://husk.w.uib.no/). From 1997 to 1999, HUSK2 was conducted as a collaborative research project between the University of Bergen, the National Health Screening Service (now part of the Norwegian Institute of Public Health), and the Municipal Health Service in Hordaland. Recruitment in HUSK2 was based on the previously conducted Hordaland Homocysteine Study in 1992–1993, in which all residents of Hordaland County born in 1925–1927 and 1950–1952 were invited to participate. In HUSK2, participants belonging to the 1925–1927 and 1950–1951 birth cohorts were reinvited [24]. In 2018, the follow-up survey, HUSK3, was initiated as a joint project between the University of Bergen, Helse Bergen HF, and the Public Dental Health Service Competence Centre for Western Norway (TkVestland). Men and women born in 1950–51, who had previously participated in HUSK2, were invited to participate in HUSK3 (https://husk.w.uib.no/husk3/).

HUSK2 is registered in ClinicalTrials.gov (Clinical trial number: NCT03013725). The HUSK surveys were conducted in accordance with the Declaration of Helsinki, and informed consent was obtained from all participants. The study protocols were approved by the Regional Committee for Medical Research Ethics (HUSK2 REK 2009/825; HUSK3 REK 2017/294). A flowchart of the HUSK study is provided in the online supplementary information Fig. 1SI.

**Fig. 1:**
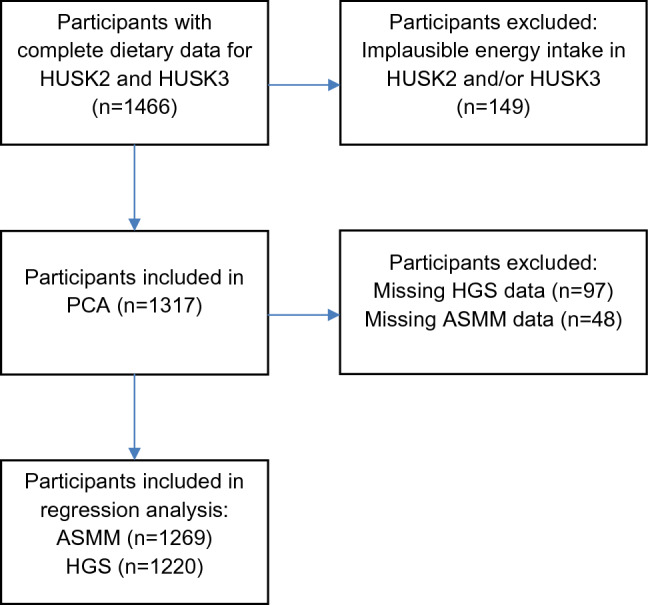
Flow diagram for participant inclusion in the principal component analysis (PCA) and regression analysis. The Hordaland Health Study 2 (HUSK2) and The Hordaland Health Study 3 (HUSK3) were conducted in 1997–1999 and 2018–2020, respectively. ASMM appendicular skeletal muscle mass; HGS handgrip strength

In the present analysis, we included participants born in 1950–1951, who completed a paper-based food frequency questionnaire (PB-FFQ) in HUSK2 (ages 46–49) and a web-based food frequency questionnaire (WebFFQ) in HUSK3 (ages 67–70), and provided complete data on body composition and handgrip strength (HGS) measured in HUSK3. Participants who reported a very low (< 3000 kJ/day for women and < 3300 kJ/day for men) or a very high energy intake (> 15,000 kJ/day for women and > 17,500 kJ/day for men) in the PB-FFQ and/or the WebFFQ were excluded (*n* = 149). After removal of participants with implausible energy intakes, 1317 participants (578 men and 739 women) were included in the PCA (Fig. 1). Of these, participants with valid data on ASMM (*n* = 1269) and HGS (*n* = 1220) were included in the regression analysis.

### Dietary assessment in HUSK2

Habitual dietary intake in HUSK2 was assessed using a 169-item semi-quantitative FFQ, developed at the Department of Nutrition, University of Oslo, Norway [25, 26]. Participants received the PB-FFQ on the day of the health survey, completed it at home, and returned it to the HUSK project centre by mail. Adequate validity has been demonstrated for the PB-FFQ in multiple studies [25–27].

The PB-FFQ inquired about intake of the following foods during the past year: bread, fat used as a spread on bread, milk as a beverage, sandwich spreads, eggs, cereals, porridge and yoghurt, coffee and tea, other non-alcoholic and alcoholic beverages, mixed dinner dishes, potatoes, rice, pasta, vegetables, sauces and condiments, cooking fat, fruits and berries, desserts, cakes, chocolate, and sweets. The PB-FFQ also assessed the use of the most common dietary supplement products in Norway and inquired about mealtime pattern. Frequency response options ranged from never to multiple times per day, per week, or per month. Portion sizes were estimated through household measures or units, e.g. number of slices/pieces, depending on the food item in question.

Information on daily intakes of foods in grams per day (g/day), energy and nutrients were generated using the software system KostBeregningsSystemet (KBS, version 3.2, database IE-96, Department of Nutrition; University of Oslo, Norway) and its associated food composition database, which is an extended version of the Norwegian Food Composition Table [28]. Calculated nutrient intakes included intake of dietary supplements. Mixed dishes were split into separate ingredients, and each ingredient was subsequently categorized into the most appropriate food group.

### Dietary assessment in HUSK3

In HUSK3, habitual dietary intake was assessed by a 279-item WebFFQ developed by the Department of Nutrition, Institute of Basic Medical Sciences, University of Oslo [29]. The WebFFQ is based on the PB-FFQ administered in HUSK2. On the day of the HUSK3 health survey, participants received information about the WebFFQ, which they were requested to complete at home and submit electronically. The WebFFQ has been validated against repeated 24-h dietary recall interviews in a subgroup of the HUSK3 study population, demonstrating reasonable ranking abilities for nearly all the included nutrients and foods [30].

The WebFFQ inquired about intake of the following foods during the past year: bread, sandwich spreads, cereals, yoghurt, non-alcoholic and alcoholic beverages, mixed dinner dishes containing meat and fish/seafood, potatoes, rice, pasta, vegetables, sauces and condiments, fruits and berries, nuts and seeds, desserts, cakes and pastries, chocolate, and sweets, as well as types of cooking fat and fat used as a spread on bread. The WebFFQ also assessed intake of dietary supplements, and an open field was included to allow participants to report foods or dietary supplements that were not included in the WebFFQ. Frequency response options were within the range of never to multiple times daily. Portion sizes were estimated by inquiring about how often a specific amount was consumed or by using images illustrating different portion sizes.

Daily intakes of foods, energy and nutrients were calculated using KostBeregningsSystemet (KBS, version 7.4, database AE18). The AE18 database is an extended version of the official Norwegian Food Composition Table, version 2018 [31]. Intake of supplements was considered in the nutrient calculations. Mixed dishes were split into separate ingredients, which allowed each ingredient to be categorized into the most appropriate food group. As the Web-FFQ used in HUSK3 was developed based on the PB-FFQ used in HUSK2, both assessment methods shared considerable similarities. However, due to a larger variety in the food market, the WebFFQ was more comprehensive than the PB-FFQ.

### Handgrip strength and skeletal muscle mass

In HUSK3, handgrip strength was measured using a Jamar + Digital Hand Dynamometer. The measurement was performed on the dominant and the non-dominant hand. Participants were seated in a straight-backed chair with feet flat on the floor. The dynamometer was held in the hand to be tested, with the upper arm placed by the side of the truncus, the forearm in neutral position, shoulders relaxed, and the elbow at a 90-degree angle. Participants were requested to squeeze the dynamometer grip handle with maximum effort for 3–5 s, while avoiding movement of other body parts. The measurement was performed three times on each hand, alternating between the left and right hand after each measurement. The test was administered by a trained dietitian, and the highest obtained measurement of HGS (in kilograms) was applied in the data analysis.

Skeletal muscle mass (SMM) was estimated by bioelectrical impedance analysis (BIA), using the SECA mBCA 515 (Seca, Hamburg, Germany). Participants were requested to stand bare feet on the device, with heels placed on the rear foot electrodes and balls of the feet placed on the front foot electrodes. Participants were asked to select a pair of hand electrodes that allowed their arms to be extended without being under strain. Once proper contact with all electrode pairs was attained, a countdown to the start of measurement was displayed on the screen. After completion of the BIA measurement, self-reported physical activity level (PAL) value (≤ 1.2 almost exclusively lying down, 1.4 almost exclusively sitting down, 1.6 mainly sitting down and occasionally standing, 1.8 mainly standing or walking, ≥ 2.0 physically strenuous), measured waist circumference and measured height were entered in the SECA mBCA 515. Using estimates of resistance (R) and reactance (Xc) derived by BIA, the equation by Kyle et al. [32] was used for estimation of appendicular skeletal muscle mass (ASMM) (kg), and subsequent calculation of the appendicular skeletal muscle mass index (ASMMI) (kg/height^2^).

### Deriving dietary pattern scores

From the PB-FFQ and the WebFFQ completed by the participants at ages 46–49 and 67–70, respectively, the average consumption of foods and beverages was estimated in g/day at each age. From the original food component variables in the raw FFQ data, 45 mutually exclusive food components that were deemed comparable between HUSK2 and HUSK3 were retained for factor analysis with PCA as the extraction method in IBM SPSS Statistics for Windows, Version 28.0. PCA was applied on the estimated average daily consumption of the 45 retained food components in HUSK3. Following visual inspection of the scree plot, three dietary patterns (eigenvalues > 1.7) were extracted using orthogonal (varimax) rotation. The scree plot is included in the online supplementary information Fig. 2SI. The identified dietary patterns were labelled on the basis of foods with an absolute factor loading ≥ 0.3 in the rotated component matrix. Dietary pattern scores (DPS) for each of the three identified dietary patterns were derived by multiplying standardized values (z-scores) of participants’ estimated consumption of each food component in g/day with the food component’s corresponding PCA factor loading (Table 2) and summing across all food components. The rotated PCA factor loadings obtained in HUSK3 were further used to calculate DPS in HUSK2 for each of the three identified dietary patterns, enhancing comparability of DPS across the two study points. The DPS reflects adherence to the dietary patterns identified by the PCA, meaning that a higher score reflects higher compliance with the dietary pattern in question. Using the DPS calculated for HUSK2 and HUSK3, an oDPS was computed for each dietary pattern, taking into account dietary intake reported in HUSK2 as well as HUSK3 (see section “Statistical analysis”).

### Statistical analysis

Normality was assessed for all continuous participant characteristic variables by visual inspection of Q-Q plots and histograms. Participant characteristics are described using summary statistics; continuous variables are presented as means (with SD). Categorical variables are presented as percentages/proportions. Differences in characteristics between men and women were assessed by the independent samples *t-*test for continuous variables and Fischer’s exact/Pearson’s Chi-squared test for categorical variables (*p* values not shown). Separate DPS for HUSK2 and HUSK3 were computed for each dietary pattern, reflecting participants’ compliance with the dietary pattern in question. Additionally, an overall DPS (oDPS) based on dietary data from both HUSK2 and HUSK3 was computed for each dietary pattern in the following manner: DPS tertile cut-points obtained for each of the three dietary patterns in HUSK3 were used to provide participants with points from 1–3 at HUSK2 and points from 1–3 at HUSK3, depending on which tertile (T) of the distribution their individual DPS were in at each study point (T1 = 1 point, T2 = 2 points, T3 = 3 points). The individual points assigned at HUSK2 and HUSK3 were then summed to create an oDPS, ranging from 2 (reflecting poor overall compliance with a dietary pattern) to 6 (reflecting high compliance with a dietary pattern). The method for computing the oDPS was adapted from a study by Robinson et al. [33].

Sex-specific multivariate linear regression models were applied to examine the association of (i) HUSK3 DPS with ASMM and HGS measured in HUSK3 (cross-sectional) and (ii) oDPS with ASMM and HGS in HUSK3 (partially longitudinal). Informed by existing evidence [17, 23, 34, 35], regression models were adjusted for a number of covariates. If entering a potential confounding factor into the regression model generated a change of > 10% in the crude estimate of association, it was included in the final model. The cross-sectional analyses were adjusted for covariates obtained in HUSK3, i.e. smoking history, self-reported physical activity, estimated total energy intake (kJ/day), and BMI (kg/m^2^), as well as educational level obtained in HUSK2. In the longitudinal approach, the regression models for oDPS were adjusted for smoking history from HUSK3, history of self-reported physical activity based on HUSK2 and HUSK3, weight change (kg) from HUSK2 to HUSK3, and educational level obtained in HUSK2, as the oDPS exposure variable incorporates dietary intake data from both time points. Smoking history was categorized into three groups: never smoked, current smoker, and previous smoker. History of self-reported physical activity was categorized into four groups: never physically active, physically active in HUSK2 only, physically active in HUSK3 only, and physically active in both HUSK2 and HUSK3. The criteria for being classified as physically active at each time point was any amount of self-reported hard physical activity per week, or ≥ 3 h of light/moderate physical activity per week. Educational level was categorized into < 12 years or ≥ 12 years of education.

All reported p-values are two-sided, and a statistical significance level of *p* < 0.05 was applied. All statistical analyses were performed using Statistical Package for Social Sciences version 28, IBM Corporation (IBM SPSS Statistics for Windows, Version 28.0. Armonk, NY: IBM Corp; 2021).

## Results

### Participant characteristics

Characteristics of the 1317 participants who were included in the PCA are presented in Table 1. Estimated protein intake adjusted for body weight (g/kg body weight) did not differ between men and women. Both men and women had mean values of maximum HGS (kg), ASMM (kg), and ASMMI (kg/m^2^) that were above the EWGSOP2 cut-offs used to define low muscle strength and muscle mass, respectively [7].

**Table 1:** Characteristics of the participants included in the principal component analysis, obtained in HUSK3 (*n* = 1317)

Characteristic^a^	All (*n* = 1317)	Men (*n* = 578)	Women (*n* = 739)
Height (cm)	171 (9.2)	179 (6.0)	164 (6.0)
Weight (kg)	76.5 (14.2)	85.1 (11.9)	69.8 (12.1)
BMI (kg/m^2^)	26.2 (4.0)	26.7 (3.4)	25.8 (4.3)
Waist (cm)	94.6 (12.4)	100.1 (10.4)	90.2 (12.1)
Total body fat mass (kg)	27.0 (8.4)	25.0 (7.8)	28.5 (8.6)
Total body fat mass percentage (%)	35.2 (8.1)	28.8 (5.9)	40.1 (5.9)
Total daily energy intake (kcal/day)	2431 (700)	2735 (694)	2193 (607)
Total daily protein intake			
*g/day*	110 (33)	122 (34)	101 (29)
*g/kg BW*	1.5 (0.46)	1.5 (0.43)	1.5 (0.48)
Maximum HGS (kg)	34.6 (10.7)	44.8 (6.8)	26.3 (4.4)
ASMM (kg)	19.1 (4.5)	23.6 (2.5)	15.7 (2.1)
ASMMI (kg/m^2^)	6.5 (1.0)	7.4 (0.7)	5.8 (0.7)
Regular, current smoking	6.3%	5.7%	6.8%
Education > 12 years^b^	47.4%	52.7%	43.8%
Leisure time moderate physical activity			
* ≥ 3 h per week*	71.8%	71.5%	72.1%
Leisure time hard physical activity			
* Any*	88.00%	90.80%	85.00%
Marital status^b^			
*Married*	78.5%	81.3%	76.3%
Medical conditions (%)			
*Diabetes*			
Currently	6.0%	8.3%	4.2%
*COPD*			
Currently	4.0%	3.8%	4.2%
*Cancer*			
Currently	2.7%	4.0%	1.6%
*Osteoporosis*			
Currently	13.1%	6.7%	18.0%
*Rheumatoid arthritis*			
Currently	3.1%	3.5%	2.8%

Slight deviations in *n* for some participant characteristics due to lack of data. *P* values for sex differences from independent samples t-test for continuous variables and Chi-square/Fisher’s exact test for categorical variables not shown

*BMI* body mass index, *kJ* kilojoule, *BW* body weight, *HGS* handgrip strength, *ASMM* appendicular skeletal muscle mass, *ASMMI* appendicular skeletal muscle mass index, *COPD* chronic obstructive pulmonary disease

^a^Continuous variables are presented as mean ± standard deviation. Categorical variables are presented as percentages.

^b^Characteristic obtained in HUSK2

Information on a range of participant characteristics was obtained from self-administered questionnaires in HUSK2 and HUSK3; the proportion of current smokers did not differ significantly between men (5.7%) and women (6.8%). A significantly greater proportion of men than women had completed > 12 years of education. Self-reported moderate leisure time physical activity did not differ significantly between men and women, with > 70% of both men and women engaging in ≥ 3 h per week. However, leisure time hard physical activity was significantly higher in men than in women. The majority of participants were married (78.5%), although the proportion of married men was significantly higher than married women. Current self-reported medication use did not differ significantly between men and women. Current presence of diabetes and cancer were reported by a significantly higher proportion of men, while osteoporosis was reported by a higher proportion of women. There were no differences in the proportions of men and women who reported chronic obstructive pulmonary disease and rheumatoid arthritis.

### Dietary patterns derived by PCA

The food components included in the PCA, as well as the rotated factor loadings for each identified dietary pattern are presented in Table 2**.** The three identified patterns explained 18% of the total variance in the dietary data. The first identified component was labelled the ‘Western’ dietary pattern, as it exhibited high positive factor loading coefficients (absolute value ≥ 0.30) for red meat and products of red meat, canned vegetables, fresh potatoes and potato chips, wholemeal bread and white bread, rice, flour and pasta, alcohol, mayonnaise, and butter. The second identified component showed high positive factor loadings for fish (fatty and lean/medium), several types of vegetables (fresh and frozen), shellfish, products and bread spreads of fish, nuts and seeds, fresh berries, citrus fruits, and eggs—labelled as the ‘Healthy’ dietary pattern. The third identified component was denoted the ‘Sweets-focused’ dietary pattern, as it was characterized by high positive factor loadings for cakes and cookies, sweet spreads, ice cream, chocolate and sweets, canned fruit, milk and cream products, apples and pears, sugar sweetened soft drinks, and brown cheese.

**Table 2:** Overview of the 45 mutually exclusive food components included in the principal component analysis, and factor loadings for the identified dietary patterns in HUSK3^a^ (*n* = 1317)

Food components	Western	Healthy	Sweets-focused
Red meat; whole, minced	**0.66**	0.22	0.05
Vegetables; canned	**0.59**	0.18	0.13
Red meat; meat and minced meat products (e.g. sausages), bread spreads of red meat (e.g. liver pate)	**0.57**	0.00	0.23
Potatoes; fresh	**0.50**	0.02	0.26
Medium brown bread, brown bread	**0.43**	-0.13	0.19
Flour, rice, pasta	**0.41**	0.20	-0.03
French fries, potato chips	**0.38**	-0.03	0.16
Beer, wine, liquor	**0.37**	0.05	-0.26
White bread	**0.35**	-0.05	0.10
Mayonnaise, dressings	**0.33**	0.05	0.10
Butter, unspecified butter/margarine	**0.33**	0.04	-0.00
Crisp bread, flatbread	-**0.30**	0.23	-0.04
Margarine, soy, hard, low-fat	0.25	-0.17	0.16
Coffee	0.22	0.05	0.07
Yoghurt	-0.15	0.13	0.05
Whole milk	0.03	0.00	0.00
Fatty fish; salmon, trout, herring, mackerel	0.12	**0.54**	-0.05
Onion, leek	0.20	**0.51**	-0.23
Other vegetables (fresh and frozen); mixed, unspecified, used in dishes with meat, bread spreads	0.08	**0.49**	-0.12
Brassica and root; carrot, swede, cabbage, cauliflower, broccoli	-0.03	**0.48**	0.04
Leafy greens; parsley, spinach, lettuce, Chinese cabbage	-0.14	**0.48**	-0.01
Lean and medium fatty fish, shellfish	0.20	**0.43**	-0.043
Beans, peas	0.17	**0.39**	-0.17
Cucumber, tomato, paprika, chilli peppers	-0.23	**0.39**	0.01
Nuts, olives, seeds	-0.12	**0.38**	0.02
Berries; fresh	-0.24	**0.36**	0.26
Citrus fruits	-0.25	**0.31**	0.25
Eggs	0.21	**0.31**	-0.01
Fish; from products, bread spreads	0.20	**0.30**	0.01
White cheese	0.10	0.29	0.17
Cooking oil	0.09	0.28	-0.08
Cream, sour cream	0.08	0.09	0.07
Cakes, cookies	0.15	-0.03	**0.53**
Jam, marmalade, honey, sweet spreads	0.15	-0.02	**0.45**
Ice cream	0.00	0.06	**0.39**
Chocolate, other sweets	0.13	-0.03	**0.33**
Fruit; canned	0.07	0.07	**0.33**
Products of milk and cream	-0.04	0.06	**0.32**
Apples, pears	-0.17	0.28	**0.31**
Sugar sweetened soft drinks	0.12	-0.14	**0.31**
Brown cheese	0.03	-0.11	**0.30**
Milk; low fat, skimmed	0.08	-0.04	0.22
Soft drinks, light	0.08	-0.04	0.18
Juice, most, smoothie	0.10	0.04	0.13
Breakfast cereals	-0.06	0.12	0.12
Individual variance explained	~ 8%	~ 6%	~ 4%
Total variance explained	18%		

^a^Factor loadings with absolute values ≥ 3 are highlighted with bold text

### Relationship between dietary pattern scores and appendicular skeletal muscle mass

Sex-specific associations of HUSK3 DPS and oDPS for each dietary pattern with ASMM at age 67–70 are presented in Table 3**.** Multivariate regression models for associations between HUSK3 DPS and ASMM were adjusted for covariates obtained in HUSK3, i.e. smoking history, self-reported physical activity, estimated total energy intake (kJ/day) and BMI (kg/m^2^), and educational level obtained in HUSK2. Multivariate regression models for associations of oDPS with ASMM were adjusted for smoking history from HUSK3, history of self-reported physical activity based on HUSK2 and HUSK3, weight change (kg) from HUSK2 to HUSK3, and educational level obtained in HUSK2.

**Table 3:** Sex-specific associations of HUSK3 dietary pattern scores and overall dietary pattern scores with appendicular skeletal muscle mass (*n* = 1269) and handgrip strength (*n* = 1220) in the Hordaland Health Study

	Appendicular skeletal muscle mass (kg)				Handgrip strength (kg)			
	Men (*n* = 548)		Women (*n* = 721)		Men (n = 543)		Women (*n* = 677)	
	Estimate (95% CI)	*p* value	Estimate (95% CI)	*p* value	Estimate (95% CI)	*p* value	Estimate (95% CI)	*p* value
**Western**								
HUSK3 DPS								
M1	0.24 (0.01 to 0.46)	0.041*	0.03 (-0.17 to 0.23)	0.759	-0.44 (-1.05 to 0.18)	0.161	0.15 (-0.29 to 0.58)	0.517
M2^a^	0.07 (-0.16 to 0.30)	0.545	0.00 (-0.18 to 0.18)	0.963	-0.65 (-1.53 to 0.24)	0.150	0.27 (-0.29 to 0.83)	0.348
Overall DPS								
M1	0.17 (-0.02 to 0.37)	0.078	-0.02 (-0.16 to 0.11)	0.720	-0.42 (-0.95 to 0.11)	0.122	0.03 (-0.25 to 0.31)	0.834
M2^b^	0.17 (-0.02 to 0.36)	0.085	-0.05 (-0.17 to 0.08)	0.473	-0.43 (-0.99 to 0.12)	0.125	0.04 (-0.24 to 0.33)	0.762
**Healthy**								
HUSK3 DPS								
M1	0.21 (0.010 to 0.41)	0.040*	0.21 (0.05 to 0.37)	0.012*	0.42 (-0.16 to 1.00)	0.151	0.18 (-0.18 to 0.53)	0.324
M2^a^	0.004 (-0.18 to 0.19)	0.969	0.12 (-0.03 to 0.27)	0.128	0.16 (-0.59 to 0.92)	0.669	0.24 (-0.23 to 0.72)	0.311
Overall DPS								
M1	0.22 (0.06 to 0.37)	0.006*	0.26 (0.14 to 0.38)	< 0.001*	0.25 (-0.17 to 0.67)	0.236	0.24 (-0.02 to 0.50)	0.074
M2^b^	0.25 (0.10 to 0.40)	0.001*	0.26 (0.15 to 0.38)	< 0.001*	0.17 (-0.26 to 0.60)	0.429	0.18 (-0.08 to 0.45)	0.169
**Sweets-focused**								
HUSK3 DPS								
M1	0.09 (-0.11 to 0.29)	0.357	0.22 (0.06 to 0.39)	0.009*	0.39 (-0.16 to 0.93)	0.164	-0.04 (-0.40 to 0.31)	0.821
M2^a^	0.13 (-0.07 to 0.33)	0.197	0.10 (-0.04 to 0.25)	0.163	0.26 (-0.49 to 1.01)	0.497	0.06 (-0.39 to 0.51)	0.800
Overall DPS								
M1	0.05 (-0.10 to 0.20)	0.543	0.11 (-0.01 to 0.22)	0.071	0.20 (-0.21 to 0.61)	0.331	0.05 (-0.20 to 0.30)	0.706
M2^b^	0.09 (-0.05 to 0.24)	0.215	0.11 (-0.001 to 0.22)	0.051	0.17 (-0.25 to 0.59)	0.423	0.03 (-0.22 to 0.29)	0.815

Estimates obtained from multivariate linear regression analysis

*DPS* dietary pattern score; *95% CI* 95% confidence interval

M1: crude, sex-specific model

M2^a^: sex-specific model adjusted for smoking history, self-reported physical activity, estimated total energy intake (kJ/day), BMI (kg/m^2^), and educational level

M2^b^: sex-specific model adjusted for smoking history, history of self-reported physical activity, weight change (kg) from HUSK2 to HUSK3, and educational level

*Statistically significant association, *p* < 0.05

The HUSK3 DPS for the ‘Western’ dietary pattern showed a significant positive association with ASMM in the crude model for men, although the association was not robust to adjustment. The HUSK3 DPS and oDPS for the ‘Healthy’ dietary pattern showed significant positive associations with ASMM in the crude models for both men and women; the association between oDPS and ASMM remained significant in both sexes after adjustment for potential confounding factors. For the ‘Sweets-focused’ dietary pattern, a significant positive association was evident between HUSK3 DPS and ASMM in the crude model for women, although statistical significance did not remain after adjustment for confounding factors.

For ASMM, the unstandardized beta coefficient of 0.25 kg in men and 0.26 kg in women for the ‘Healthy’ oDPS were the largest observed in our study, and translates to an increase of 250-g lean mass in men and 260-g lean mass in women for every unit increase in the oDPS for the ‘Healthy’ dietary pattern.

### Relationship between dietary pattern scores and handgrip strength

Sex-specific associations of HUSK3 DPS and oDPS for each dietary pattern with HGS at age 67–70 are presented in Table 3**.** Multivariate regression models for associations between HUSK3 DPS and HGS were adjusted for covariates obtained in HUSK3, i.e. smoking history, self-reported physical activity, estimated total energy intake (kJ/day) and BMI (kg/m^2^), and educational level obtained in HUSK2. Multivariate regression models for associations of oDPS with HGS were adjusted for smoking history from HUSK3, history of self-reported physical activity based on HUSK2 and HUSK3, weight change (kg) from HUSK2 to HUSK3, and educational level obtained in HUSK2. No significant associations between HUSK3 DPS or oDPS and HGS were evident for the ‘Western’, ‘Healthy’, or the ‘Sweets-focused’ dietary patterns in crude or adjusted models for men or women.

## Discussion

The current study used dietary intake data collected in middle age and old age, to examine the association of dietary pattern scores with ASMM and HGS measured in old age. We identified three distinct dietary patterns, labelled ‘Western’, ‘Healthy’, and ‘Sweets-focused’. Among the three identified dietary patterns, the oDPS for the ‘Healthy’ pattern was positively and significantly associated with ASMM measured in old age in both men and women. No associations were observed between the HUSK3 DPS or the oDPS for any of the three dietary patterns and HGS measured in old age.

Evidence pertaining to the association of dietary patterns with muscle mass and strength is characterized by discrepancies, possibly owing to the heterogeneity in study methodologies; measures of diet quality as well as measures of muscle mass and strength vary considerably between studies [23]. Additionally, dietary patterns derived by PCA are unique for the population being studied, which further limits comparability with other studies. Few studies have used dietary data collected at more than a single time point to assess the importance of à posteriori derived dietary patterns for muscle mass and strength across the lifespan.

A cross-sectional analysis within the Korea National Health and Nutrition Examination Survey, found that a PCA-derived ‘Healthy’ dietary pattern was associated with higher ASMM among men ≥ 60 years, while no association was observed between a ‘Western’ dietary pattern and ASMM [36]. While this conforms to our finding of a positive association between the oDPS for the ‘Healthy’ dietary pattern and ASMM, we did not observe a significant association between the HUSK3 DPS for the ‘Healthy’ dietary pattern and ASMM. Conversely, a longitudinal analysis in Australian men found no significant association between a PCA-derived ‘Plant-focused’ dietary pattern and skeletal muscle index, while higher scores on a ‘Traditional’ Anglo-Australian dietary pattern predicted smaller reductions in skeletal muscle index over 15 years [17]. Dietary patterns have also been investigated for an association with muscle strength. A longitudinal analysis among community-dwelling adults ≥ 60 years in Spain found no significant association of a PCA-derived ‘Prudent’ or ‘Westernized’ pattern with muscle strength [37]. This is consistent with the finding of no significant associations between either of the PCA-derived dietary patterns and HGS in our study. Conversely, Robinson et al. reported a positive cross-sectional association between a PCA-derived ‘Prudent’ diet score and HGS in women [38].

We found a positive association between the oDPS for the ‘Healthy’ dietary pattern and ASMM in our study. The ‘Healthy’ pattern was characterized by consumption a variety of fish, vegetables, nuts and seeds, fruit and berries, and eggs. The suggested mechanisms by which healthier diets may influence muscle mass and strength are numerous; such dietary patterns would expectedly provide higher intakes of a range of nutrients, e.g. vitamin D, omega-3 fatty acids and antioxidants, which have been linked to beneficial effects on muscle mass and strength in older age [39]. Evidence from nutritional interventions highlights the potential benefit of supplementation with protein, omega-3 fatty acids, vitamin D, and magnesium for promoting muscle mass and strength from middle age to older age [3]. Among the three identified dietary patterns in our study, the oDPS for the ‘Healthy’ dietary pattern exhibited the strongest positive correlations with total protein intake (g/day), protein intake adjusted for body weight (g/kg body weight), vitamin D, as well as omega-3 fatty acids (data not shown). This points towards the presence of plausible mechanistic pathways to support the significant positive association observed between the oDPS for the ‘Healthy’ dietary pattern and ASMM in our study.

Although not statistically significant in adjusted models, higher scores on the ‘Western’ and ‘Sweets-focused’ dietary patterns were positively associated with ASMM and HGS in men and women in our study. While this seems to suggest a non-intuitive favourable impact of dietary patterns characterized by consumption of discretionary foods, this may possibly be attributed to methodological aspects. In the current study, foods with absolute factor loadings of ≥ 3 were highlighted as important contributors to a dietary pattern. Using an absolute cut-off value of ≥ 3 resulted in no overlap in foods with high factor loadings across the identified dietary patterns, which in turn simplified labelling and facilitated interpretation of the dietary patterns. However, an absolute cut-off value of ≥ 2 has also frequently been used to define foods as important contributors to a dietary pattern [33, 40–42]. Positive effect estimates for the ‘Western’ and ‘Sweets-focused’ dietary patterns in our study may thus partly be explained by overlap in foods with factor loadings ≥ 2 across the dietary patterns. For instance, lean/medium fatty fish, products and bread spreads of fish, and eggs exhibited factor loadings ≥ 2 for both the ‘Healthy’ and ‘Western’ dietary pattern. Similarly, factor loadings ≥ 2 were evident for red meat products, fresh potatoes, citrus fruits, fresh berries, and low fat/skimmed milk for both the ‘Healthy’ and ‘Sweets-focused’ dietary pattern. Additionally, the ‘Sweets-focused’ pattern showed the strongest correlation with dairy products, which have been demonstrated to enhance muscle protein synthesis [3, 43].

In our cohort, 47% of the participants reported to have more than 12 years of education, while population data from Statistics Norway show that 28% among those aged ≥ 67 years have educational attainment past upper secondary school [44]. Further, only four participants (0.3%) in our cohort were found to have sarcopenia, whereas a recent meta-analysis reported an estimated sarcopenia prevalence of 14% among community-dwelling adults aged ≥ 50 years [45]. The impact of consuming a specific dietary pattern may be of greater importance to individuals with poor nutritional status compared to individuals with adequate nutritional status [46]. Hence, the lack of a consistent pattern of associations between dietary patterns and muscle mass and strength observed in our study may partly be explained by our cohort consisting of relatively healthy older adults. Additionally, the likely presence of “healthy volunteer” selection bias in the current study may limit generalizability of the findings.

As the purpose of PCA is to reduce a large number of variables into a smaller set of interpretable components that explain the largest amount of variance in the data, its ability to successfully describe dietary behaviour may be questioned [47]. However, the total explained variance of 18% in our study is in accordance with existing evidence, which establishes that PCA-extracted dietary patterns generally explain a low total percentage of variance in the complete dietary intake data [47]. Furthermore, compared with other à posteriori methods, e.g. the reduced rank regression (RRR), PCA tends to explain a greater proportion of variance in dietary intake [48]. This indicates that PCA may in fact describe existing dietary patterns in a population more accurately, although RRR-derived dietary patterns seem to be more associated with disease risk and other outcomes [49]. While PCA aims to maximize the variance explained in dietary intake, the purpose of RRR is to compute linear functions of food variables that explain the maximum amount of variance in the outcome [48]. Hence, combining different methods may be valuable in studies that aim to study real-life dietary patterns and their associations with health-related outcomes.

A strength of the current study is the number of participants with dietary data available at two time points, which allowed us to compute an overall score that reflects stability in compliance with dietary patterns from middle age to old age. Further, the comprehensive data collection in HUSK2 and HUSK3 allowed us to take into account a range of lifestyle characteristics. Adequate validity for both the PB-FFQ used in HUSK2 and the Web-FFQ used in HUSK3 has been demonstrated in previous studies [25–27, 29, 30]. Although not entirely similar, the PB-FFQ and the Web-FFQ were largely comparable in most aspects. Furthermore, objective measures of muscle mass and muscle strength were used. While magnetic resonance imaging (MRI) and computed tomography (CT) are regarded as gold standards for non-invasive estimation of muscle mass, these methods are not commonly applicable due to time and cost constraints [50]. Dual-energy X-ray absorptiometry (DXA) and BIA with high accuracy and reliability were proposed as relevant alternatives by EWGSOP2, although BIA may be preferable with regard to affordability and portability [7, 50]. Moreover, acceptable validity of BIA compared with reference methods has been demonstrated [51–53].

A limitation of the current study is that the dietary pattern scores were developed on the basis of self-reported dietary intake data, known to be prone to measurement errors [54]. However, in the context of assessing overall dietary patterns, this may be of less importance, as comparable dietary patterns have been identified with the use of different methods of dietary assessment [33]. Current evidence establishes resistance training as the most effective strategy for maintaining muscle mass and muscle strength, while the additional benefit of nutritional interventions remains uncertain [3]. Unfortunately, assessment of physical activity in HUSK2 and HUSK3 was solely based on questionnaire items and did not include objective measurement of physical activity. In addition, the items included in the questionnaire only inquired about the number of hours of moderate and hard physical activity completed per week. The use of a more comprehensive assessment of physical activity may have offered a more accurate picture of the impact of physical activity on the relationship of DPS with ASMM and HGS [17]. Furthermore, frequent collection of dietary data between middle and old age may have contributed to more precise dietary exposure variables, reflecting actual dietary choices to a greater extent. The number of subjective decisions involved in à posteriori dietary pattern analysis may further complicate the interpretation of findings [47].

Although we cannot ascertain a causal relationship between dietary behaviours and muscle mass and strength, our findings suggest that compliance through adulthood with a dietary pattern predominantly characterized by consumption of various types of fish, vegetables, nuts and seeds, fruit and berries, and eggs is associated with greater muscle mass in old age. PCA allows the identification of behaviourally meaningful dietary patterns, which offer a close relation to food-based dietary guidelines. However, using complementary methods in future studies may provide a clearer insight into how the identified dietary patterns relate to muscle health. In order to firmly establish the role of dietary patterns in the maintenance of muscle mass and muscle strength in old age, long-term longitudinal studies with repeated assessment of dietary intake are warranted.

## Data Availability

Data used in the current study are available from the corresponding author upon reasonable request.
